# The "incidental" episode of ventricular fibrillation: a case report

**DOI:** 10.1186/1752-1947-1-72

**Published:** 2007-08-30

**Authors:** Fahim H Jafary

**Affiliations:** 1 Department of Medicine, Section of Cardiology, Aga Khan University Hospital, Karachi, Pakistan

## Abstract

Polymorphic ventricular tachycardia and ventricular fibrillation (VF) carry important prognostic implications, especially in the post myocardial infarction period. However, artifact on the electrocardiographic tracing can mimic VF particularly on routinely recorded rhythm strips in hospitals. Such misinterpretation can lead to expensive (and potentially risky) diagnostic and therapeutic steps. We report on such a case and highlight the need for careful inspection of the tracing.

## Background

Arrhythmias may be documented in patients with cardiac or serious medical disorders admitted to units with telemetry monitoring, particularly intensive care wards [1]. Polymorphic ventricular tachycardia and ventricular fibrillation (VF) carry particular prognostic signficance owing to their association with sudden cardiac death. However, artifact on the electrocardiographic tracing can mimic VF particularly on routinely recorded rhythm strips in hospitals. Misinterpretation can lead to expensive (and potentially risky) diagnostic and therapeutic steps. We report on such a case and highlight the need for careful inspection of the tracing.

## Case Presentation

**Figure 1:**
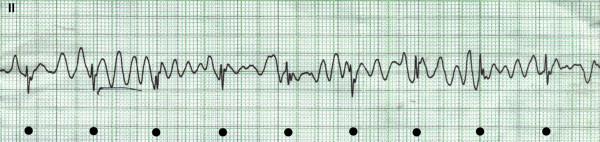
"Ventricular fibrillation" – black dots mark QRS complexes "marching through" the artifact in the background.

This 45-year-old gentleman was admitted with an acute inferoposterior myocardial infarction. Streptokinase was administered with clinical and electrocardiographic evidence of reperfusion. On the third day of admission, the following rhythm strip (figure 1) was recorded on telemetry after the alarm went off. The patient was asymptomatic and the event was documented as an episode of "transient asymptomatic ventricular fibrillation". The patient was presented on routine rounds the next morning.

Indeed, at first glance the rhythm strip appears to show ventricular fibrillation, which carries significant prognostic and therapeutic implications on the third post myocardial infarction day. On closer review, QRS complexes can be seen "marching through" the tracing (black dots), confirming that the apparent fibrillation is an artifact. Such artifacts can be induced by movement, electrical interference and lose monitor lead connections [2,3]. These electrocardiographic artifacts are not uncommon and lead to inappropriate diagnostic and therapeutic steps [4] because they tend to be misinterpreted by physicians, including cardiologists [5].

## Conclusion

Given the widespread use of telemetry monitoring in patients admitted on general medical and speciality services, artifacts on rhythm tracings will inevitably occur. Clinicians should keep such artifacts in mind when interpreting rhythm tracings depicting ventricular fibrillation, particularly when other clinical correlates of this lethal arrhythmia are absent. Careful inspection of the tracing will usually clarify the diagnosis and prevent expensive and potentially risky procedures that would otherwise follow in a genuine case. Further study is warranted to estimate the true prevalence of failure to appreciate this artifact amongst physicians of different specialties and levels of experience.

## List of Abbreviations

VF - Ventricular fibrillation.

## Competing interests

The author(s) declare that they have no competing interests.

## Authors' contributions

The author was responsible for the conception and writing of this manuscript.
